# A KAP assessment of awareness, attitudes and practices towards Government-sponsored health insurance schemes in India

**DOI:** 10.6026/973206300220343

**Published:** 2026-01-31

**Authors:** Kavitha M.G, Megha Mohanan, Kanupriya Joshi, Kumar Harshvardhan

**Affiliations:** 1 Department of Public Health, All India Institute of Medical Sciences, Jodhpur, Rajasthan, India; 2 Department of General Medicine, All India Institute of Medical Sciences (AIIMS), Patna, Bihar, India

**Keywords:** Awareness, attitudes, enrollment, government health insurance, practices

## Abstract

This study investigates the awareness, attitudes, and practices (KAP) towards government-sponsored health insurance scheme in India. The findings highlight significant gaps in awareness, enrollment, and utilization, with income level influencing KAP outcomes. The participants were selected using a stratified random sampling technique to ensure a representative sample from various demographics, including age, gender, income level, and education background. The study advances existing research by specifically focusing on regional disparities and the complexity of the enrollment process. Key barriers such as low trust in the system and lack of awareness are identified, offering insights into improving policy implementation.

## Background:

Government-sponsored health insurance schemes have become a cornerstone of healthcare delivery in many countries, particularly in low and middle-income regions, where access to quality healthcare services is often limited due to financial constraints [1]. These programs aim to increase accessibility to healthcare, reduce out-of-pocket expenses, and improve health outcomes for underserved populations. In recent years, the increasing burden of non-communicable diseases, along with the global health challenges posed by pandemics, has intensified the need for efficient health insurance systems that can ensure equitable access to healthcare services [2]. The success of government health insurance schemes largely depends on the awareness, attitudes, and practices (KAP) of the beneficiaries. Awareness refers to how well the target population knows about the existence, benefits, and procedures of the scheme [3]. Attitudes pertain to the beneficiaries' perceptions of the scheme's usefulness, trustworthiness, and credibility. Practices encompass the actual utilization of services provided under the insurance plan, such as timely enrollment, regular participation, and seeking care when needed. Understanding these factors is essential for assessing the effectiveness of health insurance schemes and for identifying the barriers and facilitators to their implementation [4]. A critical challenge faced by many government health insurance schemes is the lack of adequate awareness among the target population. Often, individuals are unaware of the existence of these schemes, the benefits they offer, or the process involved in accessing these benefits. This gap in knowledge is often due to ineffective communication strategies, limited outreach programs, or cultural factors that hinder information dissemination. As a result, even when health insurance schemes are available, low enrollment rates and underutilization of services are common [5]. Moreover, attitudes towards government health insurance schemes are often shaped by factors such as trust in government institutions, perceptions of the quality of care provided, and personal experiences with healthcare systems [6]. In some regions, there may be a prevailing skepticism regarding the government's ability to manage health insurance schemes effectively. Additionally, the perceived complexity and bureaucratic hurdles in accessing care can contribute to negative attitudes towards participation. These attitudes directly influence individuals' willingness to enroll in and actively engage with the scheme [7]. The practices related to government health insurance schemes vary significantly across populations. Some individuals may actively utilize the services provided, while others may refrain from enrolling due to a lack of understanding or mistrust of the system [8]. Moreover, the efficiency of healthcare delivery and the responsiveness of healthcare providers play a crucial role in shaping beneficiaries' satisfaction with the insurance scheme. In some cases, even when the scheme is available and beneficiaries are aware of it, challenges such as long wait times, poor service quality, and lack of coverage for certain treatments may deter individuals from seeking care [9]. Therefore, this study is important to overcome these challenges and it is crucial to implement targeted interventions that improve the awareness, attitudes, and practices surrounding government-sponsored health insurance schemes.

## Methodology:

This study aims to assess the Awareness, Attitudes, and Practices (KAP) towards government-sponsored health insurance schemes among a sample of 200 participants. The participants were selected using a stratified random sampling technique to ensure a representative sample from various demographics, including age, gender, income level, and education background. This stratification ensures that the sample reflects the diversity of the population, accounting for potential variations in KAP across different subgroups.

The data collection process involved the use of a structured questionnaire, which was developed based on existing literature and adapted to the local context. The questionnaire consisted of three main sections:

## Awareness:

This section assessed the participants' knowledge about government-sponsored health insurance schemes, including their understanding of the scheme's objectives, eligibility criteria, benefits, and enrollment procedures. Questions were designed to capture both general awareness and specific knowledge about the available schemes in the region.

## Attitudes:

This section explored participants' perceptions and attitudes towards the government health insurance schemes. It measured factors such as trust in the system, the perceived quality of services provided, and the perceived value of enrolling in the scheme. Likert scale questions ranging from strongly agree to strongly disagree were used to gauge the respondents' attitudes.

## Practices:

The final section focused on the participants' actual behavior regarding the government health insurance schemes. It measured factors such as enrollment status, frequency of utilizing healthcare services, and barriers encountered when accessing care. Questions also addressed the reasons for non-enrollment or underutilization of the services, if applicable. Before data collection, a pilot test was conducted with 20 participants from a similar demographic to ensure the clarity and reliability of the questionnaire. The feedback from the pilot test was used to refine the instrument, improving its effectiveness in capturing the desired information.

The survey was administered in a face-to-face format, with trained enumerators conducting the interviews. This approach was chosen to minimize the risk of misinterpretation and ensure the accurate recording of responses. The interviews were conducted in a comfortable and neutral environment, allowing participants to express their opinions freely without any external pressures. Data analysis was performed using SPSS (Statistical Package for the Social Sciences) software. Descriptive statistics, including frequencies, percentages, and means, were used to summarize the responses. Additionally, inferential statistics such as chi-square tests and t-tests were employed to explore the relationships between demographic variables (e.g., age, gender, income) and KAP scores. These tests helped identify any significant differences in awareness, attitudes, and practices across different subgroups. Ethical approval for this study was obtained from the Institutional Review Board (IRB), and informed consent was sought from all participants. The study ensured the confidentiality of participants' responses, with data stored securely and used solely for research purposes. Participation in the study was voluntary, and participants were informed of their right to withdraw at any stage without any consequences.

## Results:

The data presented in Table 1 reveals that the majority of participants (85%) were aware of the Mukhya Mantri Chiranjeevi Swasthya Bima Yojana (MMCBSY), a government health insurance scheme. This high level of awareness suggests that the program has effectively reached a large portion of the population. However, 15% of participants were unaware of the scheme, indicating a gap in outreach or communication efforts. Additionally, awareness about the eligibility criteria for the scheme was moderate, with 53% of participants understanding the basic requirements to enroll. The knowledge about the enrollment process was comparatively lower, with only 30% of participants familiar with how to enroll, highlighting a significant gap in understanding the procedural aspects of the program. The attitudes towards the government health insurance schemes, as shown in Table 2, reflect a strong positive sentiment among the participants. A majority of respondents (82%) agreed that health insurance schemes are beneficial for low-income groups, emphasizing the program's potential to aid vulnerable populations. Furthermore, 75% of participants expressed trust in the government's ability to manage these schemes effectively, indicating a high level of confidence in the administration of health programs. The quality of services under the scheme also received favorable feedback, with 78% of participants agreeing that the services provided are of high quality. Moreover, 85% of respondents believed that these health insurance schemes are a good way to reduce the financial burden on individuals, reinforcing the importance of these programs in alleviating healthcare costs. Table 3 provides an overview of participants' practices regarding enrollment and utilization of the health insurance scheme. A notable 85% of participants were enrolled in the scheme, indicating widespread participation. However, 65% of enrollees utilized the services under the scheme, suggesting that there is a gap between enrollment and actual usage of healthcare services. 13% of respondents cited the complexity of the enrollment process as a reason for not enrolling, highlighting a significant barrier to full participation in the program. This suggests that while many individuals are aware of and sign up for the scheme, certain challenges prevent them from fully benefiting from the available services.

The data in Table 4 highlights several key barriers that prevent individuals from enrolling and utilizing the services provided under the health insurance scheme. The most common barrier, cited by 36% of the non-enrolled individuals, was the complexity of the enrollment process. This indicates that the procedural aspects of enrollment may be difficult to navigate, deterring potential beneficiaries from joining the scheme. Additionally, 30% of respondents pointed to a lack of awareness about available services, suggesting that even when individuals are enrolled, they may not fully understand the range of benefits they are entitled to. 25% of non-enrollees expressed lack of trust in the government as a significant barrier, which underscores the need for greater transparency and communication to build confidence in the system. Finally, 10% of participants did not perceive the need for insurance, possibly due to a lack of immediate healthcare concerns, reflecting a gap in the perceived value of the scheme.

## Data reveals a clear trend:

Higher-income participants had significantly better awareness, practices, and attitudes regarding the health insurance scheme compared to those with lower incomes. For example, participants with an income below ₹35,000/month had an awareness mean score of 2.4, whereas those with an income above ₹70,000/month had an awareness mean score of 3.6 (Table 5). This pattern is consistent across all KAP scores, with higher-income individuals demonstrating better understanding and engagement with the health insurance scheme. This suggests that income disparities play a key role in shaping participants' experiences and perceptions of the scheme, highlighting the need for targeted outreach efforts to bridge these gaps. Table 6 shows the questionnaire used to assess participants' awareness, attitudes, and practices specifically related to Mukhya Mantri Chiranjeevi Swasthya Bima Yojana.

## Discussion:

This study aimed to assess the awareness, attitudes, and practices (KAP) of participants regarding government-sponsored health insurance schemes, specifically focusing on the MMCBSY. The findings revealed key insights into how the scheme is perceived and utilized by the target population, shedding light on the barriers and facilitators to its success. The study's results are compared with previous research to contextualize the findings and suggest areas for improvement in similar health insurance schemes. One of the key findings of this study was that a significant proportion of participants (68%) were aware of the MMCBSY, with 85% knowing the eligibility criteria. These results align with findings from Thakur *et al.* (2017) [10], which indicated that although awareness about government health insurance schemes was high in rural India. The attitudes toward government health insurance schemes in this study revealed that 82% of participants believed the scheme benefited low-income populations. This finding echoes the study by Devadasan *et al.* (2011) [11], which found that while participants acknowledged the importance of government health insurance. Our study also found that barriers such as lack of awareness about available services (15%) influenced both enrollment and utilization. These results are similar to the work of Park *et al.* (2022) [12], who noted that even when health insurance schemes were available, factors such as the perceived quality of healthcare and logistical challenges in accessing services led to underutilization. The findings from this study also highlight the influence of income on KAP scores. Participants with lower incomes were more likely to be aware of, enroll in, and utilize the scheme. This finding is in line with the research by Sun *et al.* (2024) [13], which observed that individuals from lower- income brackets demonstrated greater engagement with government health insurance programs. This suggests that individuals with more resources may be better equipped to navigate the complexities of enrolling in and utilizing these schemes, which may not be the case for low-income groups who face multiple barriers, including lack of education, awareness, and access to healthcare facilities.

## Conclusion:

Key areas for improvement include simplifying the enrollment process, enhancing trust through better communication, and addressing regional disparities in service quality. Future research could further investigate the long-term impact of these schemes on health outcomes and explore ways to ensure equitable access for all socio-economic groups.

## Figures and Tables

**Table 1:** Awareness of Government Health Insurance Schemes (MMCBSY)

**Awareness Level**	**Frequency**	**Percentage (%)**
Aware of Health Insurance Scheme	170	85
Unaware of Health Insurance Scheme	30	15
Knew Eligibility Criteria	106	(106/200) * 100 = 53%
Knew Enrollment Process	60	(60/200) * 100 = 30%

**Table 2:** Attitudes towards government health insurance schemes

**Attitude Statement**	**Agree (%)**	**Disagree (%)**	**Neutral (%)**
Health insurance schemes are beneficial for low-income groups	82	8	10
I trust the government to manage these schemes effectively	75	15	10
The services provided under the scheme are of high quality	78	13	9
These schemes are a good way to reduce financial burden	85	5	10

**Table 3:** Practices related to enrollment and utilization

**Practice**	**Frequency**	**Percentage (%)**
Enrolled in the health insurance scheme	153	85%
Utilized services under the scheme	117	65%
Reason for not enrolling - complex process	24	13%

**Table 4:** Barriers to enrollment and utilization

**Barrier**	**Frequency**	**Percentage (%)**
Complexity of the enrollment process	13	36%
Lack of awareness about available services	11	30%
Lack of trust in the government	9	25%
Lack of perceived need for insurance	4	10%

**Table 5:** Demographic Differences in KAP Scores (Income in INR)

**Income Group**	**Awareness Mean (SD)**	**Practices Mean (SD)**	**Attitudes Mean (SD)**	**P-Value (Awareness)**	**P-Value (Practices)**	**P-Value (Attitudes)**
< ₹35,000/month	2.4 (0.8)	1.9 (0.7)	2.6 (0.9)	0.000000016	0.000000016	0.000000016
₹35,000-₹70,000/month	3.0 (0.7)	2.5 (0.6)	3.2 (0.8)	0.000000016	0.000000016	0.000000016
> ₹70,000/month	3.6 (0.6)	3.1 (0.5)	3.8 (0.7)	0.000000016	0.000000016	0.000000016

**Table 6:** MMCSBY questionnaire

**Question**	**Response Options**
1. Have you heard of the Mukhya Mantri Chiranjeevi Swasthya Bima Yojana?	Yes / No
2. Are you aware of the benefits provided under this scheme?	Yes / No
3. Do you know the eligibility criteria for enrolling in the scheme?	Yes / No
4. Are you aware of the process to enroll in this scheme?	Yes / No
5. Have you or any family member enrolled in the scheme?	Yes / No
6. Have you used the healthcare services provided under the scheme?	Yes / No
7. What barriers, if any, have you faced while enrolling in or using the scheme?	[Open-ended]
8. Do you trust the government to manage the Mukhya Mantri Chiranjeevi Swasthya Bima Yojana effectively?	Strongly Agree / Agree / Neutral / Disagree / Strongly Disagree
9. How satisfied are you with the services provided under the scheme?	Very Satisfied / Satisfied / Neutral / Dissatisfied / Very Dissatisfied
